# Improved prediction of clinical pregnancy using artificial intelligence with enhanced inner cell mass and trophectoderm images

**DOI:** 10.1038/s41598-024-52241-x

**Published:** 2024-02-08

**Authors:** Hyung Min Kim, Taehoon Ko, Hyoeun Kang, Sungwook Choi, Jong Hyuk Park, Mi Kyung Chung, Miran Kim, Na Young Kim, Hye Jun Lee

**Affiliations:** 1 Kai Health, Seoul, South Korea; 2 Department of Medical Informatics, College of Medicine, The Catholic University of Korea, Seoul, South Korea; 3 Department of Biomedicine & Health Sciences, College of Medicine, The Catholic University of Korea, Seoul, South Korea; 4 CMC Institute for Basic Medical Science, The Catholic Medical Center of The Catholic University of Korea, Seoul, South Korea; 5 M Fertility Clinic, Seoul, South Korea; 6 Miraewaheemang Hospital, IVF Clinic, Seoul, South Korea; 7 Seoul Rachel Fertility Center, IVF Clinic, Seoul, South Korea; 8 Department of Obstetrics & Gynecology, Ajou University School of Medicine, Suwon, South Korea; 9 HI Fertility Center, Seoul, South Korea

**Keywords:** Medical imaging, Infertility

## Abstract

This study aimed to assess the performance of an artificial intelligence (AI) model for predicting clinical pregnancy using enhanced inner cell mass (ICM) and trophectoderm (TE) images. In this retrospective study, we included static images of 2555 day-5-blastocysts from seven in vitro fertilization centers in South Korea. The main outcome of the study was the predictive capability of the model to detect clinical pregnancies (gestational sac). Compared with the original embryo images, the use of enhanced ICM and TE images improved the average area under the receiver operating characteristic curve for the AI model from 0.716 to 0.741. Additionally, a gradient-weighted class activation mapping analysis demonstrated that the enhanced image-trained AI model was able to extract features from crucial areas of the embryo in 99% (506/512) of the cases. Particularly, it could extract the ICM and TE. In contrast, the AI model trained on the original images focused on the main areas in only 86% (438/512) of the cases. Our results highlight the potential efficacy of using ICM- and TE-enhanced embryo images when training AI models to predict clinical pregnancy.

## Introduction

Globally, one out of six couples suffer from infertility, but the success rate of in vitro fertilization (IVF) is still low at 20 ~ 30%^1–3^. Embryo quality is known to be one of the most important factors that contribute to a successful IVF cycle. Since single-embryo transfer (SET) is frequently performed to avoid complications resulting from multiple pregnancies, selecting the best quality embryo has become essential.

Currently, the most widely adopted method for embryo assessment is morphological evaluation by embryologists, as it is non-invasive, does not require additional instruments, and is strongly correlated with reproductive outcomes^4^. Several guidelines have been proposed to assess cleavage, morulae, and blastocysts, and the Gardner scale is typically used to evaluate blastocysts in IVF laboratories^5,6^. This scale is based on the morphology of the inner cell mass (ICM), trophectoderm (TE), and developmental stage of blastocysts. Cell structures such as the ICM and TE determine embryonic cell fate. Particularly, the ICM gives rise to the fetus and the TE forms the placenta^7^. On the Gardner scale, high-grade ICMs are prominent and easily discernible, with many cells compacted and tightly packed together. High-grade TEs are characterized by the formation of a cohesive epithelium consisting of many comprising cells.

Several studies have noted the limitations of the subjective nature of morphological scores evaluated by embryologists^8,9^. For example, several studies have reported a high degree of inter- and intra-observer variability among embryologists in scoring embryos. Furthermore, the evaluation criteria may not include all the features representative of embryo quality. Artificial intelligence (AI) techniques have recently gained attention for embryo evaluation. Embryo images captured from standard microscopes or time-lapse incubators have been used, along with relevant clinical data, to train an AI model. This model provides a predictive value for clinical pregnancy to help embryologists select embryos for transfer. Although further clinical validation is required, recent studies have reported performance levels higher than or comparable to those of human experts in predicting clinical pregnancies^10^. Additionally, age has been highlighted as an important factor in many machine learning studies^11–13^ as the natural conception rates decline as women age^14,15^. Including age in the AI model is expected to improve performance in predicting the likelihood of pregnancy.

However, the variable quality and focus of images are common pitfalls of the AI models that have been presented thus far. Most pregnancy prediction models use blastocyst images, and previous studies have established a correlation between the morphology of the ICM and TE during clinical pregnancy^16–19^. In a previous study, reasonable and stable interpretations were achieved by paying adequate attention to the ICM and TE regions^13^. In other areas which require medical image analysis, such as lung cancer or disease, segmenting the lung region from the original CT image and then predicting the presence of the cancer or asbestosis has yielded a better performance than that when using the entire original CT image^20,21^. Based on this, we hypothesized that the performance of clinical pregnancy models may be enhanced by segmentation guidance on the ICM/TE regions.

In this study, we propose a novel algorithm that could reduce the incidence of erroneous predictions by generating enhanced images from segmented ICM and TE images, which were then used to train an AI model along with female age. This is the first report to prove that the performance of the AI model can be enhanced by focusing on the ICM and TE regions. Our proposed method was validated using gradient-weighted class activation mapping (Grad-CAM), emphasizing that the ICM and TE regions are critical for predicting pregnancy. Notably, we defined a positive pregnancy indication as the presence of a gestational sac (G-sac).

## Methods

### Study design and data preparation

**Figure 1:**
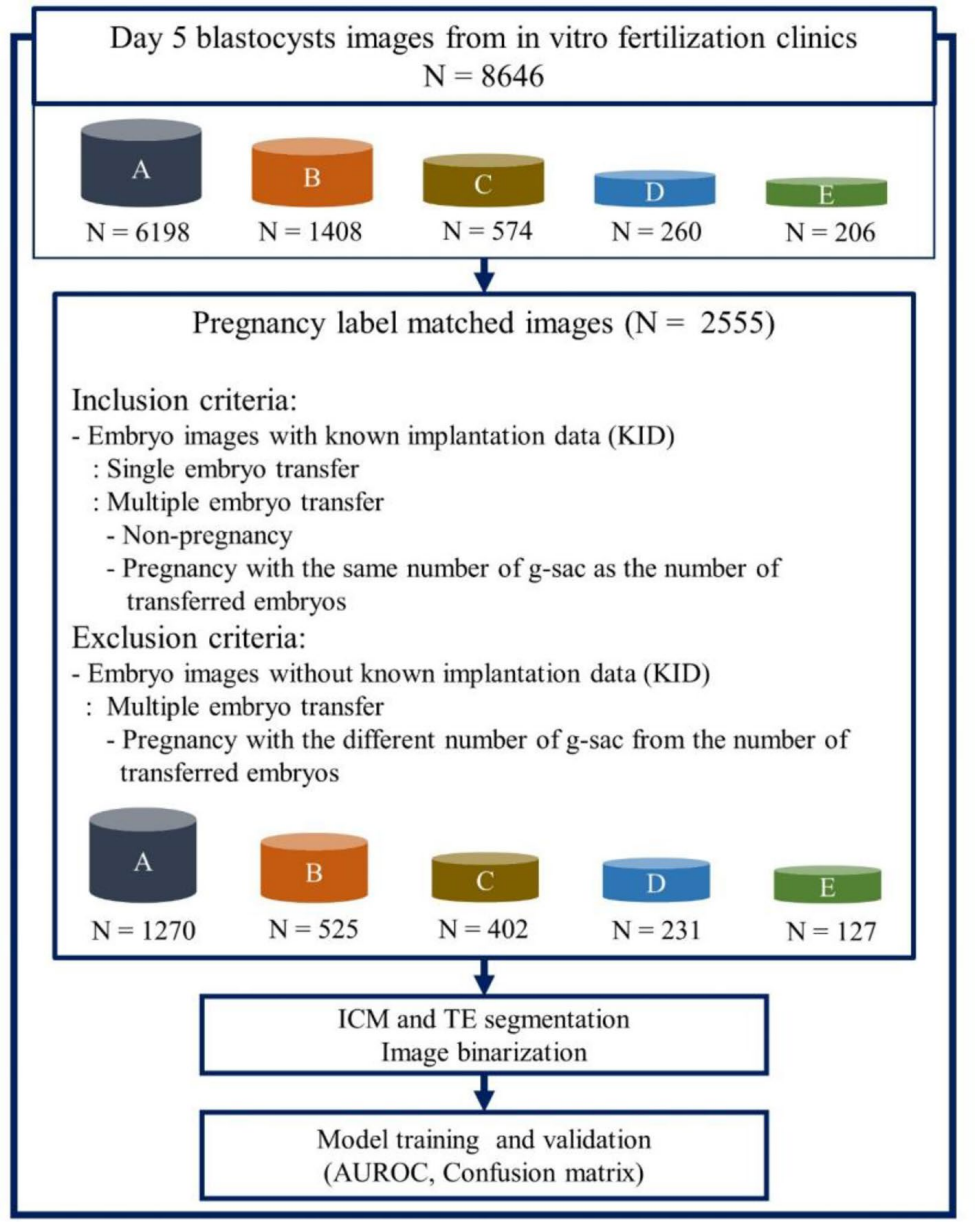
Overall process of algorithm development. *AUROC* area under the receiver operating characteristic curve, *ICM* inner cell mass, *TE* trophectoderm.

In this retrospective study conducted between June 2011 and May 2022, 8646 blastocyst images were collected on day 5 from seven IVF clinics. Images were captured using an inverted microscope or stereomicroscope before embryo transfer. Blastocysts from fresh and frozen transfers were matched to clinical pregnancy outcomes as determined by imaging the gestational sac at 4–6 weeks. The presence of a G-sac in the ultrasound scan was used to indicate clinical pregnancy. This method can be used to predict an intrauterine pregnancy with a specificity of 97.6%^22^. Additionally, using G-sac presence as an indicator can minimize non-embryonic factors being mistaken as pregnancy outcomes or advances. Blastocysts with matched pregnancy outcomes were included in the analysis. Whereas blastocysts from multiple embryo transfers with a different number of G-sacs from that of the transferred embryos were excluded. Images from clinics without relevant pregnancy information and those from the remaining clinics were divided into four groups (A, B, C, and D) with at least 200 images being provided by each group. The remaining images from the other three clinics were combined into one group (E) due to insufficient data for statistical analysis. For this study, out of a total of 8646 images, 2555 (30%) images were finally used for analysis according to the inclusion and exclusion criteria. An algorithm was developed using image pre-processing and model learning techniques. Figure 1 illustrates the overall algorithm development process and image selection criteria. The study protocol was approved by the Institutional Review Board (IRB) of the following institutions: Miraewaheemang Hospital (IRB No. 2022-RESEARCH-01), Good Moonhwa Hospital (IRB No. GMH-2022-01), HI Fertility Center (IRB No. HIRB 2022-01), Seoul Rachel Fertility Center (IRB No. RTR-2022-01), Ajou University Hospital (IRB No. AJIRB-MED-MDB-21-716), Pusan National University Hospital (IRB No. 2204-003-113), and Seoul National University Bundang Hospital (IRB No. B-2208-772-104). Informed consent was waived by the IRB of the aforementioned institutions since this study was retrospective, and the personal information in the data was blinded. The present study was designed and conducted in accordance with the relevant guidelines and regulations of the ethical principles for medical research involving human subjects, as stated by the World Medical Association Declaration of Helsinki.

### Generation of enhanced ICM and TE images

**Figure 2:**
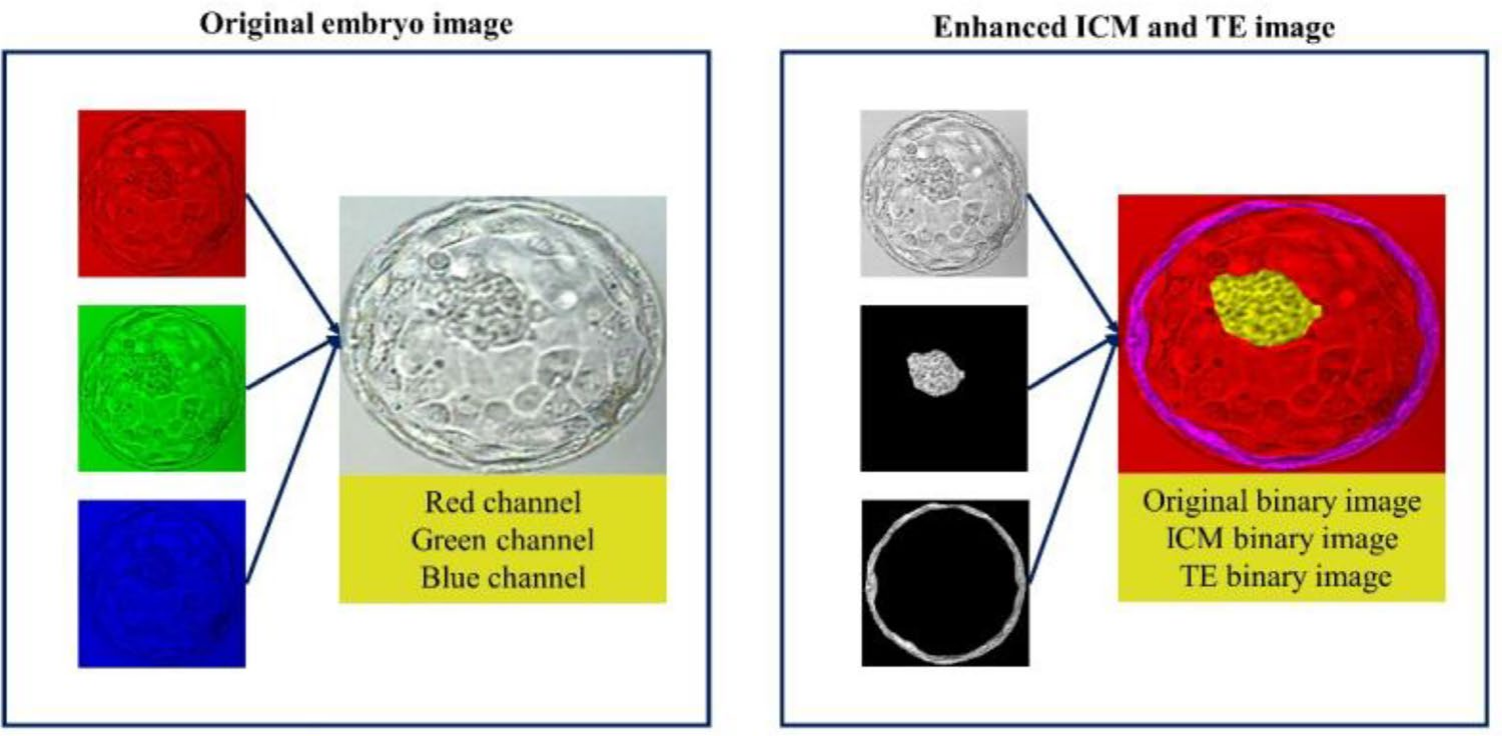
Differences in the RGB channels between the original and enhanced ICM and TE images. *RGB* red–green–blue, *ICM* inner cell mass, *TE* trophectoderm.

The day-5-blastocyst embryo images collected from the IVF clinics were manually annotated by trained personnel to identify the ICM and TE regions. The ICM was defined as the tightly packed mass of cells within the inner blastocoel cavity and the TE was defined as the spherical layer of outer cells surrounding^23^. The accuracy of the annotation was manually examined first by embryologists and then by a group of laboratory directors with over 20 years of experience. The coordinates of the ICM and TE regions were stored as JSON files, and the Python library OpenCV (version 3.3.1) was then employed to generate segmented images of the ICM and TE regions from the original images. The original embryo image was represented by merging the red, green, and blue channels to display the natural colors. Conversely, the grayscale image was composed of only a single channel. Enhanced ICM and TE images were created by combining three grayscale images: the original embryo, ICM, and TE images. Instead of using the original red, green, and blue channels found in a regular color image, each image was converted into a grayscale and treated as a single channel. These three grayscale images were then merged to form the final enhanced ICM and TE images using the three channels. Conventional convolutional neural networks were designed to process images with three channels, and grayscale images were merged into three channels to align them with the original image format (Fig. 2).

### Training data split and image preprocessing

Two separate experiments were conducted to evaluate the performance of the proposed method. In the first experiment, pregnancy was predicted using only the original embryo microscopy images. In the second experiment, we predicted pregnancy after generating enhanced images using the ICM and TE segmentation images. In total, 2555 images were divided into either training data (2043 images, 80%) or model performance test data (512 images, 20%). We then utilized threefold cross-validation to divide the 2043 images, including as the training data, into three folds. Each fold was utilized to train and validate the model, and performance was evaluated using fixed model performance test data. Supplementary Table S1 illustrates the overall data segmentation and distribution. Before applying the enhanced ICM and TE images to the deep-learning model, image pre-processing was performed to make them suitable for training. During the image pre-processing step, all pixel values of the image were normalized, and the image was resized to 224 × 224 pixels. Since the sample size of the image dataset was not sufficiently large, we attempted to improve the efficiency of image classification by applying image augmentation^24,25^. This made the model more robust by augmenting the images with transformations, allowing it to learn more images during training. To perform image augmentation, TensorFlow 2.10.0, a deep-learning neural network API in Python^26^, was used with the following options:

- Random brightness
- Random saturation
- Random contrast
- Flip images vertically
- Flip images horizontally

In addition, since a pre-trained model was used, the image was resized to fit the learned size of each pre-trained model. All data split and image preprocessing procedures described above were applied to both the first and second experiments.

### Statistical analysis

To determine whether there was a significant difference in age between the negative and positive pregnancy groups, a t-test was performed on the entire dataset. Furthermore, binary classifiers were used to estimate the probability of an instance belonging to the positive class using prediction scores; as these scores are often poorly calibrated, they may not accurately reflect the true probabilities^27^. The Hosmer–Lemeshow test was used to compare the expected and observed frequencies of the positive class to assess calibration^28^.

### Model development and evaluation

**Figure 3:**
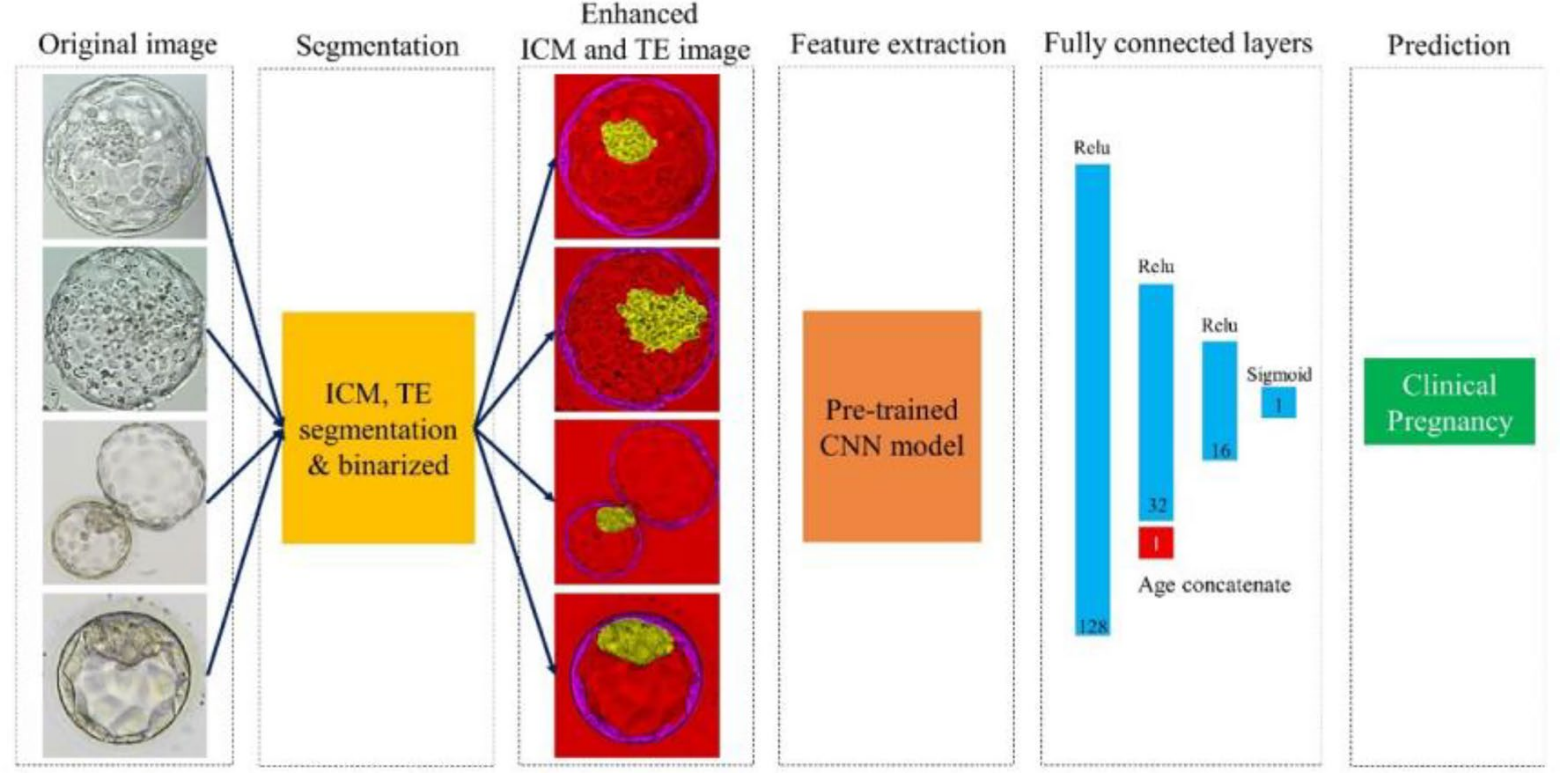
Overall process of the prediction algorithm using images of the gestational sac. *ICM* inner cell mass, *TE* trophectoderm, *CNN* convolutional neural network.

For the convolutional neural network model, we utilized the original and enhanced ICM and TE images and compared their respective performances. Due to the limited sample size, we fine-tuned the pre-trained model to learn the model and used 224 × 224 images, which is the same size as that utilized in ImageNet. Moreover, after concatenating the patient’s age to the last fully connected layer of the architecture, it was configured to return the predicted pregnancy value through the sigmoid layer. The complete process from image input to predicted value return is illustrated in Fig. 3. Model training was conducted on a machine equipped with an Intel Xeon CPU @ 2.10 GHz and an RTX3090 (24 GB) GPU, utilizing the Python programming language (version 3.8.0).

To evaluate the performance of our algorithm, we used four key metrics: sensitivity, specificity, accuracy, and the AUROC. The formulas for these metrics are as follows.

- TP = number of true positive samples.
- TN = number of true negative samples.
- FP = number of false positive samples.
- FN = number of false negative samples

$${\text{Sensitivity}}=\frac{TP}{TP+FN}$$

$${\text{Specificity}}=\frac{TN}{TN+FP}$$

$${\text{Accuracy}}=\frac{TP+TN}{TP+FP+TN+FN}$$

The areas under the receiver operating characteristic curves (AUROCs) of the AI models were compared using original embryo images and enhanced ICM and TE images. We utilized three representative pre-trained models for the convolutional neural network architecture, DenseNet121^29^, VGG16^30^, and ResNet50^31^. These models had been trained from large-scale ImageNet datasets^24^ and were fine-tuned to assess our embryo images. Grad-CAM is a technique used in the field of computer vision to understand and visualize important image regions, contributing to deep neural network prediction. It aims to generate a heat map that highlights the input image regions that are the most important for a particular class prediction. By generating these heat maps, Grad-CAM provides insights into the internal workings of deep neural networks, allowing researchers and practitioners to interpret and understand the features learned by the network. In this study, two embryologists with more than 20 years of expertise as laboratory directors visually inspected the Grad-CAM images and confirmed whether the AI model exhibited a higher focus on the ICM and TE regions when utilizing enhanced ICM and TE images.

## Results

A t-test results, as shown in Table 1, indicated that the average age of the negative group (36.2 years) was significantly higher than that of the positive group (34.0 years). This age difference was also observed for each of the five clinics, with the negative group consistently having a higher average age than that of the positive group; this difference was statistically significant for all five clinics.

**Table 1:** Baseline distribution of embryos and female patient ages in each IVF laboratory.

Laboratory	Negative pregnancy		Positive pregnancy		*P*-value
	N	Mean ± SD	N	Mean ± SD	
Total	1743	36.2 ± 3.9	812	34.0 ± 3.1	< 0.001
A	931	36.3 ± 4.0	339	33.4 ± 2.9	< 0.001
B	309	35.8 ± 4.3	216	34.0 ± 3.2	< 0.001
C	325	36.5 ± 3.1	77	34.7 ± 3.0	< 0.001
D	95	36.6 ± 3.6	136	35.6 ± 2.9	0.027
E	83	35.3 ± 4.3	44	33.5 ± 3.8	0.023

*IVF* in vitro fertilization, *N* sample size, *SD* standard deviation.

**Figure 4:**
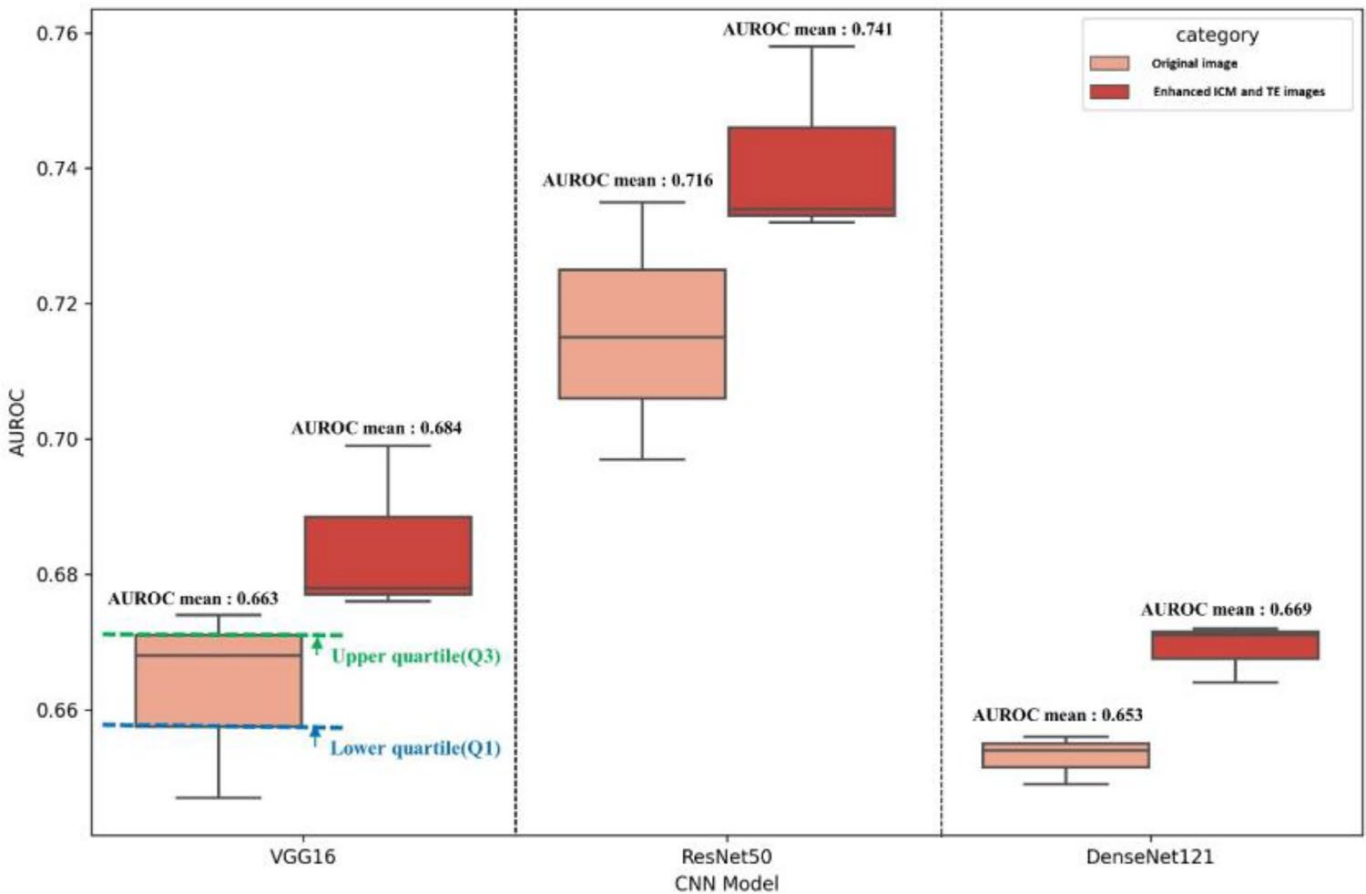
AUROC box plot comparison of the AI models. *AUROC* area under the receiver operating characteristic curve, *AI* artificial intelligence, *ICM* inner cell mass, *TE* trophectoderm, *Q* quartile.

**Figure 5:**
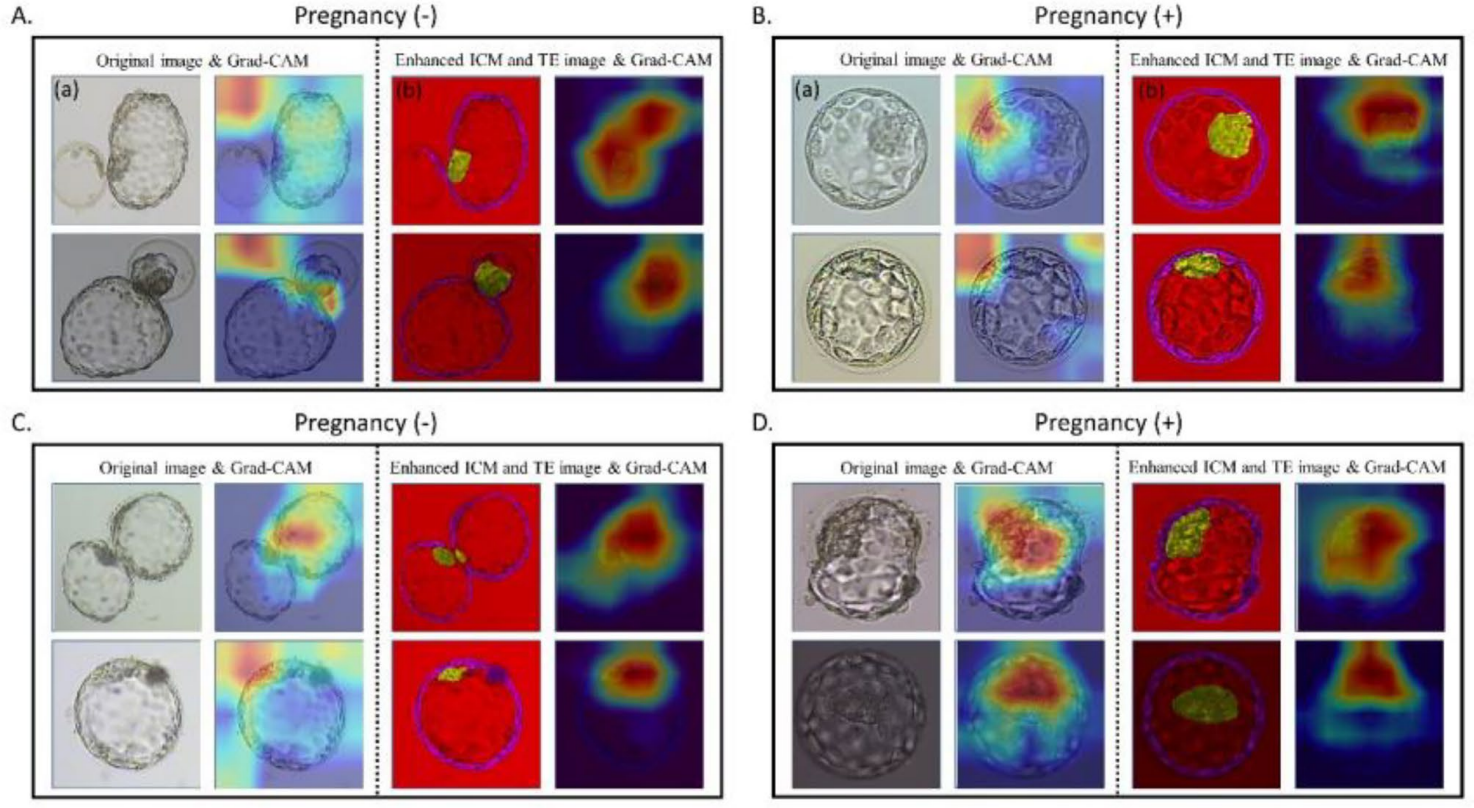
Grad-CAM results. (**A**) For a negative pregnancy, the original image (a) led to an incorrect prediction, while the enhanced ICM and TE image (b) produced a correct prediction. (**B**) For pregnancy positivity, when the original image was incorrectly predicted (a), the enhanced ICM and TE image (b) were correctly predicted. (**C**) Both the original and the enhanced ICM and TE images were incorrectly predicted as negative pregnancies. (**D**) Both the original and the enhanced ICM and TE images were incorrectly predicted as pregnancy-positive. *ICM* inner cell mass, *TE* trophectoderm, *Grad-CAM* gradient-weighted class activation mapping.

Our results revealed that all three models performed better when trained using enhanced ICM and TE images with age information. The best performance was obtained when utilizing the enhanced ICM and TE images in the ResNet50 architecture, with an average mean (standard deviation) AUROC of 0.741 (0.014) (Table 2). AUROCs’ performance by threefold is shown in Supplementary Fig. S1. The original image also exhibited the best performance in the ResNet50 architecture, with an AUROC mean and variance of 0.716 (0.019). The boxplots in Fig. 4 show that the enhanced ICM and TE images had better AUROC values than that of the original images across all three models. The lower quartile (Q1) of the enhanced ICM and TE images was higher than the upper quartile (Q3) of the original images, indicating better performance based on the AUROC metric. In our study, we analyzed the regions that the CNN model learned from using Grad-CAM when predicting pregnancy with the proposed reconstructed image and the original image. Our findings indicated that the ICM and TE regions were learned more intensively when using the reconstructed images instead of the original images. The Grad-CAM results in Fig. 5A,B illustrate a case where the original image led to an incorrectly predicted outcome, while the respective reconstructed image led to a correct prediction. In the original image, the features from the ICM and TE regions could not be extracted, leading to an incorrect prediction. Conversely, the reconstructed image model was trained to focus on the ICM and TE regions, which produced an accurate prediction. However, in the case of Fig. 5C,D, the features in the ICM and TE regions within the embryo could be adequately identified in both the original and reconstructed images; however, the predicted outcome was still incorrect.

**Table 2:** Performance of deep learning models for G-SAC prediction.

	CNN Model	AUROC	Accuracy	Sensitivity	Specificity
Original image	VGG16	0.663 (0.014)	0.652 (0.008)	0.690 (0.048)	0.636 (0.025)
	ResNet50	0.716 (0.019)	0.663 (0.012)	0.705 (0.067)	0.645 (0.020)
	DenseNet121	0.653 (0.004)	0.663 (0.013)	0.684 (0.049)	0.654 (0.033)
Reconstructed image	VGG16	0.684 (0.013)	0.654 (0.005)	0.760 (0.042)	0.609 (0.020)
	ResNet50	0.741 (0.014)	0.682 (0.015)	0.714 (0.042)	0.669 (0.028)
	DenseNet121	0.669 (0.004)	0.669 (0.024)	0.694 (0.033)	0.648 (0.032)

*G-SAC* gestational sac, *CNN* convolutional neural network, *AUROC* area under the receiver operating characteristic curve.

We checked Grad-CAM for 4 out of a total of 512 cases from the test set: when both the original and enhanced ICM and TE images were correctly predicted (n = 314), when both were incorrectly predicted (n = 106), when only the original image was correctly predicted (n = 35), and when only the enhanced ICM and TE images were correctly predicted (n = 57). The number of images focused on learning the ICM and TE regions increased from 85.5% (438/512) to 98.8% (506/512) when the images were enhanced using ICM and TE (Table 3). In Table 3, “Other” refers to cases where the model focused on other areas not including any ICM or TE areas.

**Table 3:** Grad-CAM results.

	Original image Grad-CAM focus		Enhanced ICM and TE image Grad-CAM focus	
	ICM/TE N (%)	Other N (%)	ICM/TE N (%)	Other N (%)
Both images correct N = 314	270 (86.0%)	44 (14.0%)	312 (99.4%)	2 (0.6%)
Both images incorrect N = 106	93 (87.7%)	13 (12.3%)	102 (96.2%)	4 (3.8%)
Only the original image correct N = 35	29 (82.9%)	6 (17.1%)	35 (100.0%)	0 (0.0%)
Only enhanced ICM and TE images correct N = 57	46 (80.7%)	11 (19.3%)	57 (100.0%)	0 (0.0%)
Total N = 512	438 (85.5%)	74 (14.5%)	506 (98.8%)	6 (1.2%)

*Grad-CAM* gradient-weighted class activation mapping, *ICM* inner cell mass, *TE* trophectoderm.

The Hosmer–Lemeshow test was conducted on our best-performing model to assess calibration and provided non-significant results with a p-value < 0.001 and a Brier score of 0.241. To address this issue, we applied isotonic regression and achieved a well-calibrated model with a p-value of 0.265 and a Brier score of 0.178. A calibration plot is shown in Supplementary Fig. S2.

## Discussion

This study aimed to determine the effect of segmentation guidance on AI performance in predicting clinical pregnancy using embryo images. AI predictive performance improved when the model was guided by ICM and TE segmentation. Although overlooking appropriate areas in embryo images has been previously pointed out as an issue of AI, there have been no reported attempts to solve it. To the best of our knowledge, this is the first study to verify improvements in AI predictive performance using ICM and TE segmentation.

Segmentation technology is commonly employed in various medical imaging domains. The utilization of segmentation technology in the computer-aided diagnosis of medical images is increasingly recognized as a valuable and critical aspect of medicine. This approach allows for the effective utilization of large volumes of medical data while mitigating the risk of misdiagnosis resulting from subjective visual observations^32–34^. Research in other medical imaging areas has indicated that AI trained on datasets simplified through segmentation has a higher diagnostic performance when focusing on the regions of interest^21,35,36^. Although the operating mechanism of deep learning algorithms remains elusive, it is widely accepted that the less complex the data, the higher the learning efficiency that AI can achieve under the same conditions^37^.

The ICM and TE morphologies have long been used worldwide to evaluate and screen embryos. Since these structures play important roles in determining the fate of cells during embryonic differentiation, many studies have investigated the correlation between their morphology and pregnancy rates^16,18^. Several studies have shown that the ICM and TE are also strongly related to the live birth rate, and the ICM is known as a major factor that can predict euploid embryos^19,38^. Despite expecting the AI model to learn and accurately represent the morphology of ICM and TE by training it with segmented images the results were not as good as anticipated. Particularly, when explaining the inferences of the deep learning model using techniques such as Grad-CAM, we noticed that the model often failed to focus on the ICM or TE. However, the enhanced ICM and TE images proposed in this study ensured the morphology of such embryonic structures was emphasized for the deep learning model. This approach provided a strong basis for creating an AI system that can predict clinical pregnancy by incorporating relevant clinical domain knowledge.

One of the major strengths of this study was the high-quality dataset. The key to developing an effective AI model is to train it with a sufficient volume of good data. This study used nationally curated data that were well-refined and labeled from embryo images collected from multiple institutions, which were endorsed by a third-party inspector. Since the dataset was created as part of a government-funded project, quality control was adequately performed, and the original dataset is scheduled to be made public in the future, ensuring external reliability.

The additional task of investigating the incorrectly predicted cases was undertaken in this study, particularly in the four categories shown in Table 3. Two laboratory directors closely examined the original and enhanced ICM and TE images. For clear images, both the original model and the enhanced ICM and TE models correctly predicted the actual outcome. For less clear images, the segmentation model outperformed the original model. For inherently messy images, both models failed to predict pregnancies. Of the 75 messy images, 25 showed irregular contours of the zona pellucida, embedded sperm in the zona, or cytoplasmic darkness, and the AI may have misinterpreted these characteristics as fragmentation. The use of well-focused and clear images ensures a fair performance of the AI model. Furthermore, image reconstruction using ICM and TE segmentation was validated and may be helpful for less-clear images to a certain extent. However, for cases where the original model outperformed the segmentation model, no specific pattern was found in Grad-CAM, and potential explanations include variables such as clinical or genetic information that may help overcome the limitations of morphological assessment. Moreover, we provided the AI model with the correct ICM and TE information according to the labeling of the laboratory directors, but Grad-CAM confirmed that the model had a bias to learn by looking at other regions besides ICM and TE. However, it answered correctly in two cases and incorrectly in four cases (Table 3). The incorrect answers can be interpreted as the failure to extract features from ICM and TE, and the two correct answers can be interpreted as features found elsewhere inferring probabilities similar to the embryo's state by chance. In conditions where prediction is challenging, such as pregnancy, the accuracy of the prediction is typically around 70%. In the 44 cases within the “Other” group the original image was supposed to focus on a different region (other than the ICM or TE); however, the correct answer was given. This is because Grad-CAM can learn the wrong region of an image and sometimes as a result the AI model's predicted value can coincidentally match the correct answer.

In our study, we applied isotonic regression to solve the calibration problem. However, even after applying isotonic regression, the true positive rate decreased in the range of high predicted probabilities (0.7–0.9). This has been confirmed to be due to the occurrence of incorrect cases. In these cases, the AI model predicted good-quality embryos after being successfully trained on ICM and TE images; however, pregnancy did not occur due to various complex factors. It is important to note that calibration can be more difficult for small sample sizes because there may be insufficient information to accurately estimate the mapping between the predicted scores and probabilities. In such cases, it may be necessary to collect additional data or employ alternative methods to enhance calibration. Therefore, we aim to collect a global dataset that includes both domestic and foreign data and develop a more accurate and robust model in future research.

While morphology evaluation is the most widely used methodology for embryo selection, chromosomal analysis, also known as preimplantation genetic testing (PGT), provides additional information. However, PGT is invasive and expensive, and there is no established way to interpret and resolve the problem of potential mosaicism^39^. To overcome this challenge, AI technologies have been proposed as a non-invasive alternative. Static and time-lapse images of blastocysts can predict ploidy with AUC values of 0.68–0.76^40–42^. However, this level of accuracy is insufficient compared to invasive PGT. Further research is required to improve the accuracy. This should include the application of the technology proposed in this study and the combination of morphological evaluation by AI with metabolomic analysis and non-invasive PGT using culture media or blastocysts.

This study had limitations. To generalize our results, an international study is required, as our study was conducted on the same racial background. In addition, because our dataset consisted of images from multiple institutions, the color and sharpness of the images varied slightly. Although this allows for better performance in terms of validation compared to training a single institution, it is still difficult to guarantee the same performance when unfamiliar, heterogeneous images are processed. Furthermore, to automate our method, we must develop an algorithm that automatically segments the ICM and TE areas. In future research, we plan to develop an ICM and TE segmentation model that automatically segments these areas from the original embryo image, creates an enhanced ICM and TE image, and feeds it as input to the AI model. Moreover, the blastocyst stage and ICM and TE grade of the embryo are known to be major factors affecting pregnancy. Therefore, evaluating these factors in addition to image interpretation would improve prediction accuracy. However, the grading of ICM and TE is subjective and requires expert help to obtain information. Therefore, we were unable to reflect this information in our study. In future studies, researchers should create a prediction model by distinguishing blastocyst stages and conduct ongoing research on the impact of such factors on actual pregnancy.

In conclusion, this study demonstrated that the predictive performance of AI improved when using enhanced ICM and TE images. AI can provide objective information to empower the assessment of embryologists; however, it often analyzes irrelevant parts of images, leading to incorrect results, particularly when the image is out of focus. Our research findings may help generalize the AI model for application to embryo images with various focuses. Further research can be the first step towards transparent AI models for embryo assessment.

### Supplementary Information

Supplementary Information.

## Data Availability

All data information can be accessed through ‘AI-Hub’ (www.aihub.or.kr).
