# Supplementary material for: Improved prediction of clinical pregnancy using artificial intelligence with enhanced inner cell mass and trophectoderm images
Source: Sci Rep. 2024 Feb 8;14:3240. doi: 10.1038/s41598-024-52241-x (PMC10853203; doi:10.1038/s41598-024-52241-x)
Supplement: Supplementary file 1 — Supplementary Information. [file 41598_2024_52241_MOESM1_ESM.docx]

**Improved Prediction of Clinical Pregnancy Using Artificial Intelligence with Enhanced Inner Cell Mass and Trophectoderm Images**

Hyung Min Kim, Ph.D.,^a^ Taehoon Ko, Ph.D.,^b^ Hyoeun Kang, M.S., ^a^ Sungwook Choi, M.D.,^c^ Jong Hyuk Park, Ph.D.,^d^ Mi Kyung Chung, Ph.D.,^e^ Miran Kim, M.D., Ph.D.,^f^ Na Young Kim, M.D.,^g^ Hye Jun Lee, M.D.^a,*^

^a^ Kai Health, Seoul, South Korea

^b^ Department of Medical Informatics, College of Medicine, The Catholic University of Korea, Seoul, South Korea

^c^ M Fertility Clinic, Seoul, South Korea

^d^ Miraewaheemang Hospital, IVF clinic, Seoul, South Korea

^e^ Seoul Rachel Fertility Center, IVF clinic, Seoul, South Korea

^f^ Department of Obstetrics & Gynecology, Ajou University School of Medicine, Suwon, South Korea

^g^ HI fertility center, Seoul, South Korea

**Table S1.** Overall dataset split ratio and 3-fold cross-validation set composition

| Fold 1  (n=2043) | Fold 2  (n=2043) | Fold 3  (n=2043) | Test  (n=512) |
| --- | --- | --- | --- |
| Validation  (n=681) | Train  (n=681) | Train  (n=681) | Test |
| Train  (n=681) | Validation  (n=681) | Train  (n=681) | Test |
| Train  (n=681) | Train  (n=681) | Validation  (n=681) | Test |


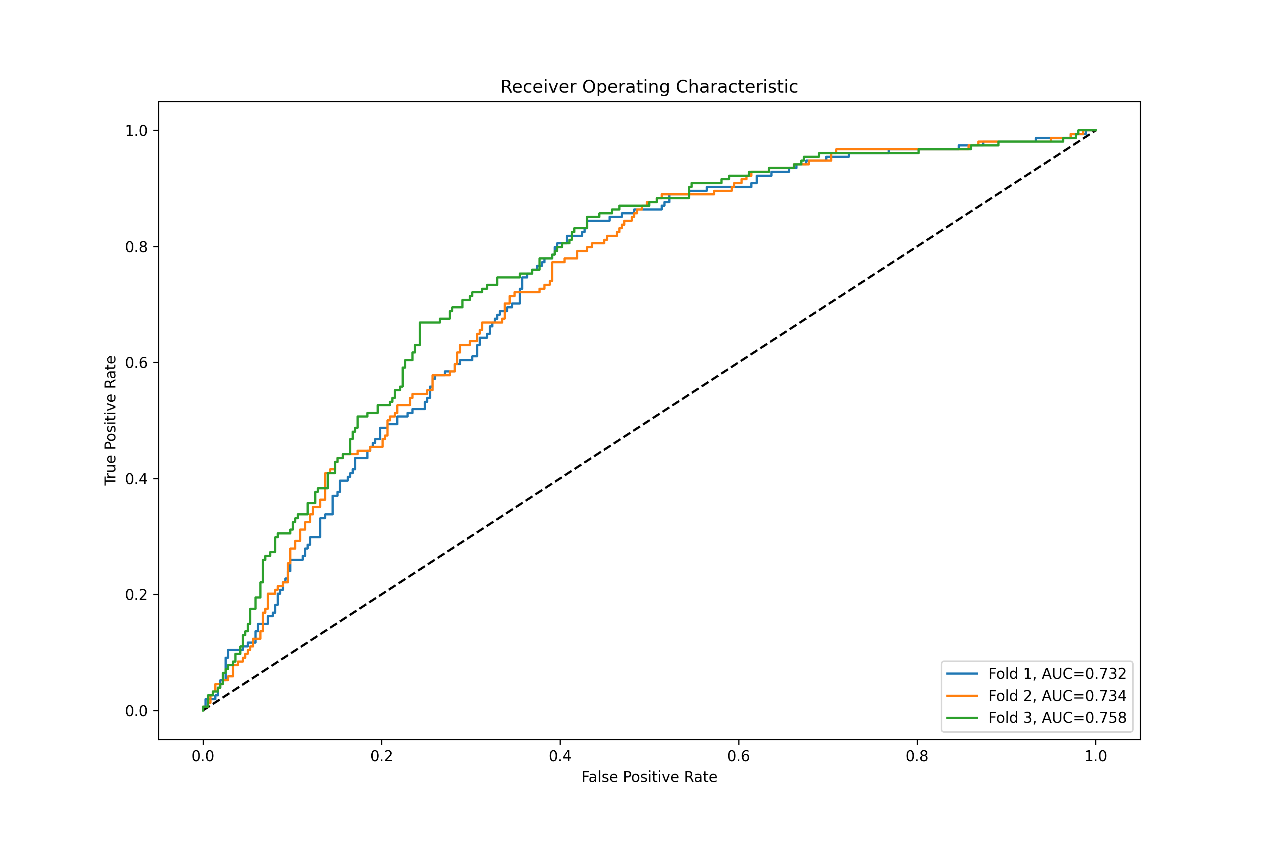


**Figure S1.** AUROCs of best performance model. AUROC, area under the receiver operating characteristic curve.


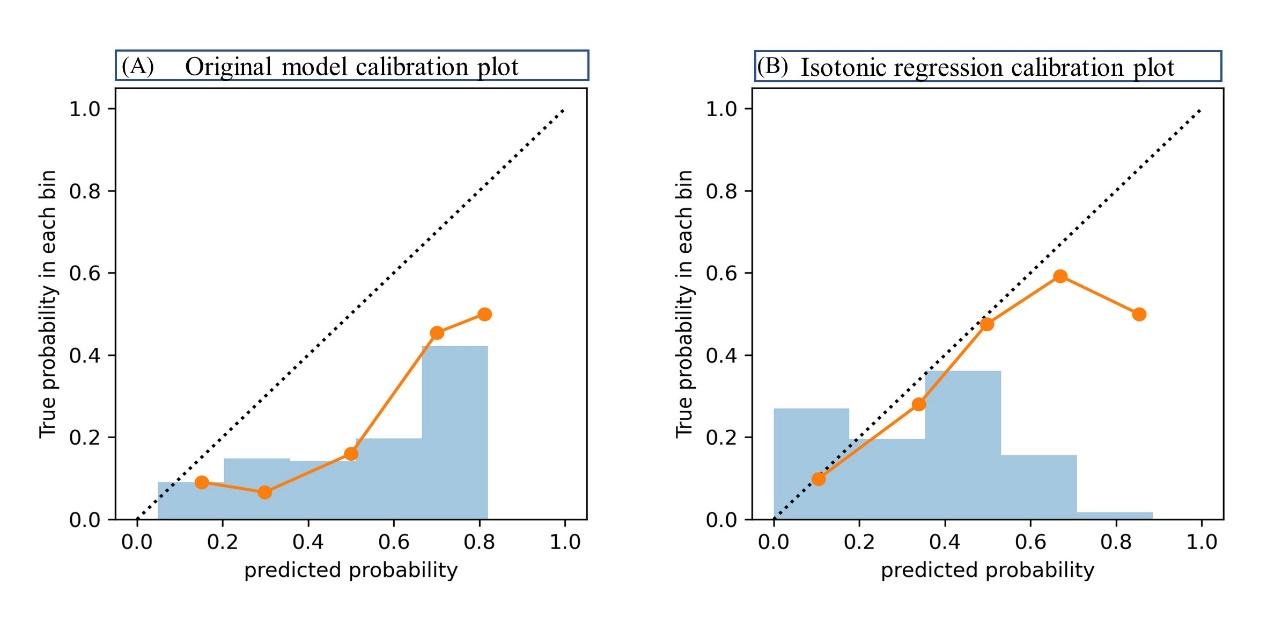


**Figure S2.** Calibration results of the original and the isotonic regression models. (A) Hosmer–Lemeshow test results: X-squared = 152.75, P-value < 0.0001, Brier score: 0.241; (B) Hosmer–Lemeshow test: X-squared = 3.9723, P-value = 0.2645, Brier score: 0.178.
